# Exploring the Molecular Basis for Substrate Affinity and Structural Stability in Bacterial GH39 β-Xylosidases

**DOI:** 10.3389/fbioe.2020.00419

**Published:** 2020-05-15

**Authors:** Mariana Abrahão Bueno de Morais, Carla Cristina Polo, Mariane Noronha Domingues, Gabriela Felix Persinoti, Renan Augusto Siqueira Pirolla, Flávio Henrique Moreira de Souza, Jessica Batista de Lima Correa, Camila Ramos dos Santos, Mário Tyago Murakami

**Affiliations:** ^1^Brazilian Biorenewables National Laboratory, Brazilian Center for Research in Energy and Materials, Campinas, Brazil; ^2^Brazilian Synchrotron Light Laboratory, Brazilian Center for Research in Energy and Materials, Campinas, Brazil

**Keywords:** glycoside hydrolase family 39, structural stability, substrate specificity, X-ray crystallography, *Xanthomonas citri*

## Abstract

The glycoside hydrolase family 39 (GH39) is a functionally expanding family with limited understanding about the molecular basis for substrate specificity and extremophilicity. In this work, we demonstrate the key role of the positive-subsite region in modulating substrate affinity and how the lack of a C-terminal extension impacts on oligomerization and structural stability of some GH39 members. The crystallographic and SAXS structures of a new GH39 member from the phytopathogen *Xanthomonas citri* support the importance of an extended C-terminal to promote oligomerization as a molecular strategy to enhance thermal stability. Comparative structural analysis along with site-directed mutagenesis showed that two residues located at the positive-subsite region, Lys166 and Asp167, are critical to substrate affinity and catalytic performance, by inducing local changes in the active site for substrate binding. These findings expand the molecular understanding of the mechanisms involved in substrate recognition and structural stability of the GH39 family, which might be instrumental for biological insights, rational enzyme engineering and utilization in biorefineries.

## Introduction

Bacterial glycoside hydrolase family 39 (GH39) members typically act as β-xylosidases (Vocadlo et al., 2002; Czjzek et al., 2005; Corrêa et al., 2012), but recent studies have shown that some GH39 enzymes can be devoid of catalytic activity against small xylose-based substrates (Ali-Ahmad et al., 2017). Instead, such enzymes are polyspecific (Jones et al., 2017) and involved in biofilm formation (Baker et al., 2015). These findings demonstrate a more diverse functional role of bacterial GH39 members than previously envisaged and open new questions regarding the molecular basis for substrate specificity in this family. Moreover, the existence of few GH39 structurally characterized members in CAZy database (Lombard et al., 2014) reveals that this family is still poorly explored and mechanistic details for substrate binding remain mostly unknown.

By elucidating the crystal structure of the mesophilic GH39 enzyme from the oligotrophic bacterium *Caulobacter crescentus* (Santos et al., 2012), it was verified a C-terminal shortening in comparison to thermophilic GH39 members (Yang et al., 2004; Czjzek et al., 2005), which precluded the formation of tetramers. However, the scarce in solution and structural information for other members of the family remained inconclusive whether there is a correlation between the C-terminal extension and extremophilicity.

The plant pathogen bacterium *Xanthomonas citri* is the causative agent of the citrus canker and contains a broad arsenal of glycoside hydrolases for hemicellulose degradation including endo-β-1,4-xylanases, β-xylosidases, arabinofuranosidases, acetyl xylan esterases, and α–glucuronidases. Besides being of industrial interest, these enzymes play important roles in virulence and survival of *Xanthomonas* species (Rajeshwari et al., 2005; Szczesny et al., 2010; Déjean et al., 2013). Among these enzymes the XacXynB, a putative β-xylosidase belonging to GH39, was found in the genome of *X. citri* (Silva et al., 2002) and according to its primary sequence it presents the C-terminal shortening as observed to *C. crescentus*, CcXynB2 (Santos et al., 2012).

Herein, we describe the structural and functional characterization of the GH39 XacXynB, contributing with new structural determinants for substrate recognition in its family, based on site-directed mutagenesis and molecular dynamics simulations, and supporting a role of the extended C-terminus in extremophilicity, combining phylogenetic analysis with high resolution structure determination.

## Materials and Methods

### Molecular Cloning and Site-Directed Mutagenesis

XacXynB gene (GenBank: AAM38893.1) from *X. citri* codifies a 502 amino-acid residues mature protein with homology to the GH39 family. The signal peptide was removed and a 6xHis-tag was inserted, using the pET28a vector aiming further purification steps. The mutants K166D and D167G were generated by site-directed mutagenesis. The amount of 30 μmol of the designed primers (K166D_R: 5′ gcgttctcccagaaatcatccagattgggctcgttcc and K166D_F: 5′ ggaacgagcccaatctggatgatttctgggagaacgc; D167G_R: 5′ cggcgttctcccagaaccccttcagattgggctc and D167G_F: 5′ gagcccaatctgaaggggttctgggagaacgccg) were added to 20.8 ng⋅μL^–1^ of pET28a-XacXynB with 0.5 mM dNTPs, 1 μM Mg(SO_4_)_2_, 5 μL Platinum^®^ Pfx DNA Polymerase 10× buffer and 1.25 U Platinum^®^ Pfx DNA Polymerase (Life Technologies, Carlsbard, CA, United States). The annealing and extension temperatures were 55°C during 60 s and 68°C for 7 min, respectively. The procedure was repeated by 18 cycles. In order to digest methylated parental DNA, the reaction was incubated with 1 U *Dpn*I (Fermentas, Waltham, MA, United States), 37°C during 1 h. The reaction product was transformed into competent *Escherichia coli* DH5α cells and after growing, plated into 2% *(v/w)* Luria-Bertani containing 50 μg⋅mL^–1^ kanamycin and 50 μg⋅mL^–1^ chloramphenicol. After 16 h growing at 37°C, the clones DNA were extracted and submitted to Sanger sequencing.

### Protein Expression and Lysis

All plasmids were transformed into thermocompetent BL21(DE3)ΔSlyD cells with pRARE2 plasmid and grown in LB containing 2% *(v/v)* of inoculum, 50 μg⋅mL^–1^ kanamycin and 50 μg⋅mL^–1^ chloramphenicol at 37°C, 4 h at 200 rpm until A_600 nm_ reached 0.7. The induction with 0.1 mM Isopropyl β-D-1-thiogalactopyranoside was performed at 20°C for 16 h. The cells were collected by harvesting at 5000 × *g* and resuspended in lysis buffer (20 mM sodium phosphate, pH 7.5, 500 mM NaCl, 5 mM imidazole, 1 mM phenylmethylsulfonyl fluoride and 5 mM benzamidine), and disrupted by lysozyme treatment (80 μg⋅mL^–1^, 30 min, on ice), followed by sonication (50% amplitude and 6 pulses of 15 s on ice using a tip 406) in a Vibracell VCX 500 device (Sonics and Materials, Newtown, CT, United States). The extract was harvested at 30,000 × *g* and filtered.

### Purification Steps

All purification steps were performed using a Fast Performance Liquid Chromatography System (GE Healthcare, Little Chalfont, United Kingdom). The crude extract was applied, at 1 mL⋅min^–1^ flow rate, into a 5 mL HiTrap Chelating column (GE Healthcare, Little Chalfont, United Kingdom) charged with 100 mM NiSO_4_ and pre-equilibrated with 20 mM sodium phosphate, pH 7.5, 500 mM NaCl and 5 mM imidazole. The extract was washed with 10 column volumes and eluted in a 0–100% non-linear gradient with a buffer containing 500 mM imidazole. The following step of size-exclusion chromatography was performed in a HiLoad Superdex 75 16/60 colunm (GE Healthcare, Little Chalfont, United Kingdom) pre-equilibrated with 20 mM sodium phosphate, pH 7.5, 150 mM NaCl with a 0.5 mL⋅min^–1^ flow rate.

### Circular Dichroism

A purified XacXynB sample was analyzed by far-UV circular dichroism (CD) measurements (195–260 nm), at a protein concentration of 0.2 mg⋅mL^–1^ in 20 mM of sodium phosphate pH 7.4. CD measurements were carried out using a JASCO 815 spectropolarimeter (JASCO Inc., Tokyo, Japan) equipped with a Peltier temperature control unit using a 0.1 cm path quartz cuvette. The solvent spectra were subtracted in all experiments to eliminate background effects. The CD spectrum was the average of 20 accumulations using a scanning speed of 100 nm min^–1^, spectral bandwidth of 1 nm, and response time of 0.5 s. The thermal denaturation of the enzyme was characterized by measuring the ellipticity changes at 222 nm induced by a temperature increase from 20 to 100°C, at a heating rate of 1°C⋅min^–1^.

### Crystallization

Crystallization experiments were carried out using the sitting-drop vapor-diffusion method. For initial screening, 0.5 μL of the protein solution at a concentration of 30 mg⋅mL^–1^ in 20 mM Tris–HCl (pH 7.5, 150 mM NaCl) was mixed with an equal volume of the screening solution and equilibrated against a reservoir containing 80 μL of the latter solution at 18°C. Single crystals appeared in a condition consisting of 0.1 M MIB Buffer (pH 5.0) and 25% *(w/v)* PEG1500 after 5 days.

### X-Ray Diffraction Data Collection and Processing

Crystals were transferred into a cryosolution consisting of the reservoir solution supplemented with 20% *(v/v)* glycerol and were flash-cooled in a nitrogen-gas stream at 100 K for data collection. X-ray diffraction data were collected at the MX2 beamline (LNLS, Campinas, Brazil) with the radiation wavelength set to 1.459 Å. The images were collected using an oscillation angle of 1° and an exposure time of 30 s per image. The MAR Mosaic 225 mm (MAR Research, Norderstedt, Germany) charge-coupled device (CCD) was used to record the intensities. Data were processed using the XDS package (Kabsch, 2010).

### Structure Determination and Refinement

The crystal structure of XacXynB was solved by molecular replacement (MR) calculations through the program PHASER (McCoy et al., 2007) using CcXynB (PDB entry: 4EKJ) as template. The best MR solution required extensive manual chain tracing, mainly of the C-terminal subdomain. Refinement cycles were carried out with REFMAC5 (Murshudov et al., 1997) or PHENIX refine (Afonine et al., 2012). After each cycle of refinement, the model was inspected and manually adjusted to correspond to computed σ_A_-weighted (2*F*_*o*_–*F*_*c*_) and (*F*_*o*_–*F*_*c*_) electron density maps using the program COOT (Emsley and Cowtan, 2004). The water molecules were manually added at positive peaks above 2.0 σ in the difference Fourier maps, taking into consideration hydrogen-bonding distances. The refined structure was evaluated using the program MolProbity (Chen et al., 2010).

### Small Angle X-Ray Scattering

SAXS measurements were performed at the SAXS1 beamline (LNLS, Campinas, Brazil). The radiation wavelength was set to 1.488 Å and X-ray scattering was recorded using a Pilatus 300 K (Dectris, Baden, Switzerland). The sample-to-detector distance was adjusted to a scattering-vector range of 0.01 < *q* < 0.25 Å^–1^, where *q* is the magnitude of the *q-*vector defined by *q* = 4πsinθ/λ (2θ is the scattering angle). 10 frames of 30 s each were recorded to monitor radiation damage. Buffer scattering was recorded and was subtracted from the protein scattering. The integration of SAXS patterns was performed using FIT2D (Hammersley et al., 1997). Data were analyzed using the GNOM program (Svergun, 1992). The radius of gyration (*R*_g_) was estimated by the indirect Fourier transform method and the distance distribution function P(r) was calculated from the scattering curve using the maximum diameter (D*_max_*) as a parameter. The molecular envelopes were calculated from the experimental SAXS data using the program DAMMIN (Svergun, 1999). Ten runs of *ab initio* shape determination yielded highly similar models [normalized spatial discrepancy (NSD) values of <1], which were then averaged using the DAMAVER package (Volkov and Svergun, 2003). The crystallographic structure was fitted into the SAXS molecular envelope using the program SUPCOMB (Kozin and Svergun, 2001).

### Phylogenetic Analysis

The full-length sequences of characterized GH39 enzymes described in CAZy database were retrieved along with XacXynB. A multiple sequence alignment was performed using MAFFT v7.299b (Katoh et al., 2002) and a Maximum Likelihood phylogenetic tree based on the WAG matrix-based model was constructed using FastTree v.2.1.8 software (Price et al., 2010). The phylogenetic tree was visualized and annotated using the iTol web tool.

### Enzyme Assays

XacXynB (WT and mutants) was incubated with 2 mM of 4-nitrophenyl β-D-xylopyranoside (Sigma-Aldrich, St. Louis, MO, United States) and the product release was quantified at 400 nm. To determine the optimum pH and temperature profiles, enzymatic reactions were carried out at different pHs using McIlvaine (McIlvaine, 1921) buffers (pH 3.5–8.0, with 0.5 steps) and at a temperature range of 15–75°C (with 5°C steps). The absorbance values were converted in relative activity. These assays were performed in triplicate. Kinetic characterization was performed in a substrate range of 0.1–25 mM (4-nitrophenyl β-D-xylopyranoside), 50 mM McIlvine buffer pH 5.5 (McIlvaine, 1921), with 13.6 μM of enzyme at 40°C during 15 min. Kinetic parameters, *K*_M_, *V*_max_, *k*_cat_, and *k*_cat_/*K*_M_, were calculated from initial velocities.

### Capillary Zone Electrophoresis

The cleavage of xylooligosaccharides (XOS) from xylobiose (X2) to xylohexaose (X6) was analyzed at pH 5.5 at 40°C. Aliquots were taken after 16 h of reaction and heated to 95°C for 15 min for enzyme inactivation. For labeling, samples were incubated with 20 μL μM citric acid (2.5 M), 8 μL sodium cyanoborohydride (1 M) and 1.5 μL APTS (100 mM). The samples were incubated at 80°C for 2 h, dried and then resuspended in 90 μL of running buffer (40 mM potassium phosphate pH 2.5). The derivatized sugars were analyzed in a P/ACE MDQ instrument (Beckman Coulter, Brea, CA, United States) equipped with a laser induced fluorescence detection module. A capillary of 50 μm internal diameter and 20 cm effective length was used. Electrophoretic conditions were set to 20 kV/70–100 mA with reverse polarity at 25°C. APTS-labeled sugars were excited at 488 nm and emission was detected at 520 nm. The electrophoretic behavior of labeled products was compared to standards.

### Molecular Dynamics Simulations

Molecular dynamics simulations were performed with WT XacXynB, and K166D and D167G mutants. Mutations were introduced manually by replacement of the residues from WT crystal structure before simulations. A His-tag fragment (present in the crystal structure) from residues Gly-5 to His2 was removed prior to the simulations. The protonation state of all residues was assigned considering neutral pH. Protonation of His residues were further assessed according to their chemical environment. The inferred acid/base residue (Glu162) was protonated since its pKa was approximately 9 according to the program PROPKA (Li et al., 2005). Simulations were performed using Amber18 software (Case et al., 2018) with the FF14SB force-field (Maier et al., 2015) for the protein and the TIP3P force-field (Jorgensen et al., 1983) for water molecules. The systems were minimized, keeping the protein fixed and then the entire systems have been allowed to relax. The systems were heated in steps (to 100, 200, and 300 K) in the NVT ensemble at intervals of 50 ps. Spatial restraints were applied to the entire protein during the first heating interval (to 100 K), being all restraints released after this step. The density was converged up to water density at 300 K during 100 ps in the NPT ensemble. Runs of 100 ps were performed to equilibrate the root-mean-square deviation (RMSD) in the NVT ensemble with a time step of 1 fs. The time step was subsequently increased to 2 fs, employing the SHAKE algorithm (Ryckaert et al., 1977). Simulations have been extended to 100 ns (production). Analysis of the trajectories has been carried out using standard tools of AMBER and VMD (Humphrey et al., 1996).

## Results

### C-Terminal Region Affects Oligomerization and Structural Stability

**FIGURE 1:**
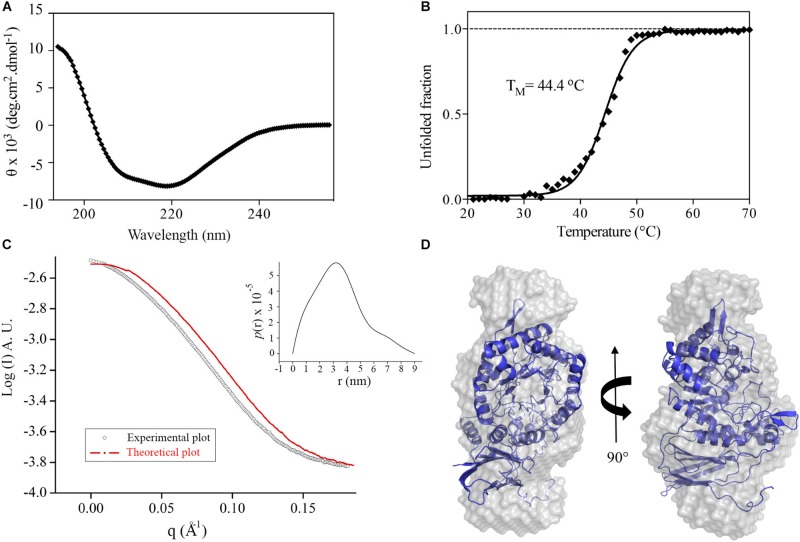
XacXynB secondary structure, stability and oligomeric state. **(A)** Circular dichroism spectrum of XacXynB. **(B)** Thermal denaturation profile of XacXynB monitored at 222 nm by circular dichroism spectroscopy. The midpoint transition temperature (*T*_M_) was calculated from the sigmoidal fit of the denaturation curve. **(C)** SAXS profile compared to the theoretical curve based on the crystallographic model of XacXynB. The pair-distance distribution function plot, P(r), is shown in the *inset*. **(D)** The crystallographic structure of XacXynB fitted into the SAXS envelope at two orientations.

The recombinant GH39 enzyme XacXynB was heterologously expressed in a folded and stable form with a typical CD spectrum of α/β proteins (Figure 1A), which is in accordance with the conserved fold present in GH39 β-xylosidases (Yang et al., 2004; Czjzek et al., 2005; Santos et al., 2012). Despite of the modular structural organization with an ancestral accessory domain, the thermal unfolding occurred following a two-state model with a midpoint transition temperature (T_M_) of 44°C (Figure 1B). This T_M_ is similar to that observed for other glycoside hydrolases from *X. citri* (Santos et al., 2014; Domingues et al., 2018).

The oligomeric state of XacXynB in solution was determined by SAXS and resulted in a radius of gyration (R_g_) of 2.72 ± 0.01 nm. The distance distribution [P(r)] profile indicates an elongated shape of XacXynB (Figure 1C) with maximum dimensions compatible with a monomeric state. The *ab initio* SAXS modeling resulted in a molecular envelope with a low NSD with the crystallographic monomer (Figure 1D), corroborating the monomeric form of XacXynB in solution. The low-resolution structure and SAXS parameters are similar to those observed for the monomeric GH39 member from *C. crescentus* (CcXynB) (Santos et al., 2012).

**FIGURE 2:**
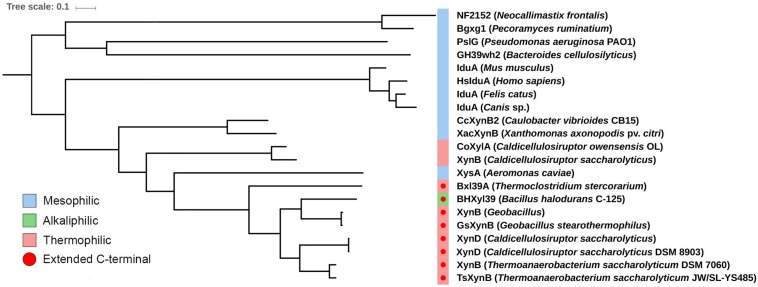
Evolutionary relationship of the C-terminal extension in mesophilic and extremophilic GH39 members. Maximum likelihood phylogenetic tree of characterized GH39 enzymes annotated in CAZy database (Lombard et al., 2014), highlighting the mesophilic, alkalophilic and thermophilic organisms. The C-terminal extension (red circle) is only observed in enzymes from alkalophilic or thermophilic organisms. All nodes have support values >0.7.

Phylogenetic analysis considering all characterized GH39 enzymes available in the CAZy database revealed a correlation between the presence of an extended C-terminus and microorganism extremophilicity (Figure 2 and Supplementary Figure 1). While most GH39 members from extremophilic bacteria contain a C-terminal extension, those adapted to milder conditions systematically lack this motif. It indicates that oligomerization via C-terminal extension might be an important and conserved adaptation in this family to extreme environments.

The XacXynB structure was elucidated at 1.7 Å resolution with one full-length protomer in the asymmetric unit (Table 1). It clearly reveals the lack of C-terminal extension in comparison with termophilic GH39 structures, from *Thermoanaerobacterium saccharolyticum* (TsXynB) (Yang et al., 2004) and *Geobacillus stearothermophilus* (GsXynB) (Czjzek et al., 2005; Supplementary Figure 2A). In homotetrameric GH39 β-xylosidases, the C-terminal extension is swapped between two monomers, forming the main dimer-stabilizing interface, named interface II (Yang et al., 2004; Supplementary Figures 2B,C).

**TABLE 1:** Data collection and refinement statistics of XacXynB.

**Data collection**	
Space group	*C*2
**Cell dimensions**	
a, b, c (Å)	117.14; 57.09; 81.12
α, β, γ (°)	90; 107.5; 90
Molecules per AU	1
Resolution (Å)	38.67–1.71
Observed reflections	328,998 (29,274)
Unique reflections	54,724 (5,211)
CC_1__/__2_^†^	0.999 (0.848)
I/σ(I)	20.09 (2.47)
Completeness (%)	98.21 (94.01)
R_meas_	0.058 (0.610)
Multiplicity	6.0 (5.6)
**Refinement**	
Resolution (Å)	28.93–1.71
Number of reflections	54,710
R_work_/R_free_	0.163/0.197
Number Of protein residues	506
**B-factor (Å^2^)**	
Average	23.80
Macromolecules	22.80
Solvent	31.26
**Root mean square deviations**	
Bond lengths (Å)	0.007
Bond angles (°)	1.17
**Ramachandran plot**	
Favored (%)	98
Allowed (%)	1.8
Disallowed (%)	0.2
Molprobity clashscore	1.49
Protein Data Bank code	6UQJ

*Values in parentheses are for highest-resolution shell. ^†^CC_*1*/*2*_: correlation between intensities from random half-datasets (Karplus and Diederichs, 2012).*

Although the other structural elements (including the dimeric interface I) are conserved in XacXynB, the main dimer-stabilizing interface (type II) is missing due to the lack of the long C-terminus. Crystal packing and energetic analyses by PDBePISA (Krissinel and Henrick, 2007) did not indicate any energetically stable interface for XacXynB oligomers, supporting the monomeric state observed in solution.

### Active Site Mapping Indicates Room for Accommodation of Xylooligosaccharides With Distinct Degrees of Polymerization

**FIGURE 3:**
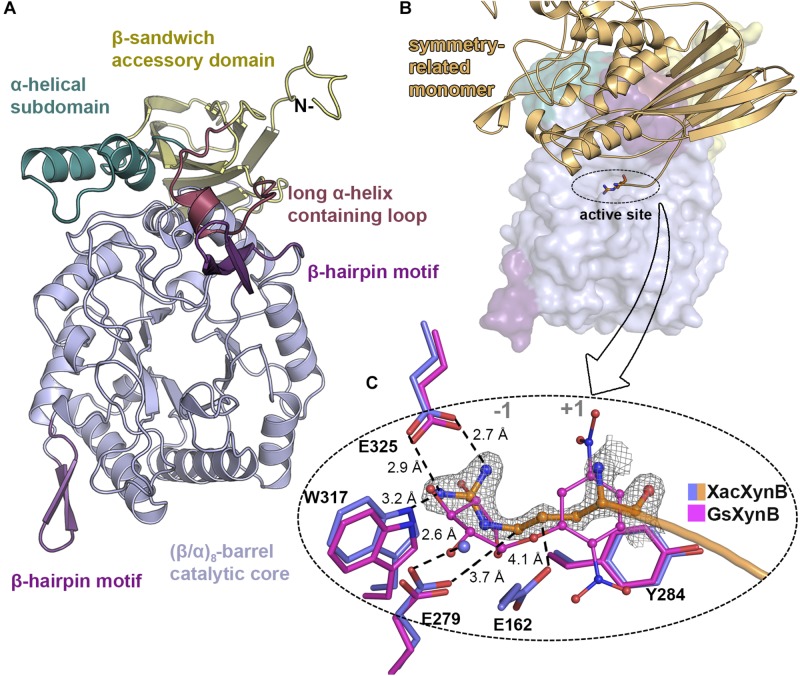
XacXynB crystallographic structure and active site mapping. **(A)** Cartoon representation highlighting domains and motifs by different colors. **(B)** Surface representation with the symmetry-related molecule (orange cartoon) blocking the active-site cleft. The arginine from the His-tag is represented as sticks. **(C)** Zoom into active site (–1 and +1 subsites regions indicated) highlighting the interactions of the arginine from the symmetry-related molecule (with orange carbon atoms) with the residues Glu325, Trp317, Glu279, and Glu162 (purple carbon atoms) and water (purple sphere). The complex of the GH39 member from *Geobacillus stearothermophilus* mutant E160A (GsXynB; PDBID 2BFG) with 2,5-dinitrophenyl-β-D-xyloside (pink carbon atoms) was superimposed on the XacXynB structure, allowing the comparison of the xylose position in the –1 subsite. The 2F_o_–F_c_ electron density map (contoured at 1.0 σ level) of the N-terminal arginine residue is represented.

XacXynB conserves canonical elements of GH39 members, with (α/β)_8_-barrel catalytic core decorated with two extra β-hairpin motifs (between the secondary elements of the barrel β_3_-α_3_ and β_6_-α_6_) and a C-terminal β-sandwich accessory domain with an α-helical bundle (residues 433–466) (Figure 3A). The ancestral accessory domain is not independent from the catalytic domain being formed additionally by a β-strand from the very N-terminus and tightly associated with the α_6_ and α_7_ secondary structural elements from the barrel. Similar to CcXynB, XacXynB contains a long α-helix-containing loop (residues 389–406) between the β-strands 14 and 15 of the accessory domain, which links the accessory domain and the parental catalytic core (Figure 3A). This feature is not observed in any other structurally characterized GH39 members (except CcXynB and XacXynB) conferring a very distinctive catalytic interface. The α-helical bundle and long α-helix-containing loop from the accessory domain along with the β_6_-α_6_ β-hairpin are critical to stabilize the conformation of loops forming the substrate binding subsites.

**FIGURE 4:**
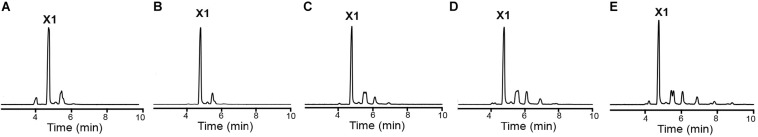
XacXynB activity on xylooligossacharides from X2 to X6. The cleavage pattern was analyzed by capillary zone electrophoresis after reactions with **(A)** X2, **(B)** X3, **(C)** X4, **(D)** X5, and **(E)** X6. The released products were mainly xylose (X1).

Due to crystal packing, the N-terminus of the symmetry molecule is occupying the entire active-site cleft, inserting the side chain of an arginine in the −1 subsite (Figure 3B). This arginine residue (Arg-1) makes part of the synthetically introduced sequence of a 6xHis-tag in the protein and occupied exactly the position of a xylosyl moiety in the −1 subsite interacting with Glu325, Trp317, and Glu279 (Figure 3B). The binding mode of this peptide at the active site along with structural comparisons with the GsXynB complex with 2,5-dinitrophenyl-β-D-xyloside allowed to identify putative key residues involved in substrate binding such as Phe118, Phe168, Tyr230, Trp317, Glu325, and Phe337 in the −1 subsite and Tyr284 in the +1 subsite, beyond the catalytic residues Glu162 (acid/base) and Glu279 (nucleophile) (Vocadlo et al., 1998; Bravman et al., 2001; Yang et al., 2004; Figure 3C). Based on these structural analyses, it would be expected that the active site of XacXynB could accommodate xylooligosaccharides with different degrees of polymerization, which was further confirmed by capillary zone electrophoresis experiments. The enzyme was able to hydrolyze xylooligosaccharides from X2 to X6, producing xylose as the final product (Figure 4).

### Positive-Subsite Region Modulates Substrate Affinity

**FIGURE 5:**
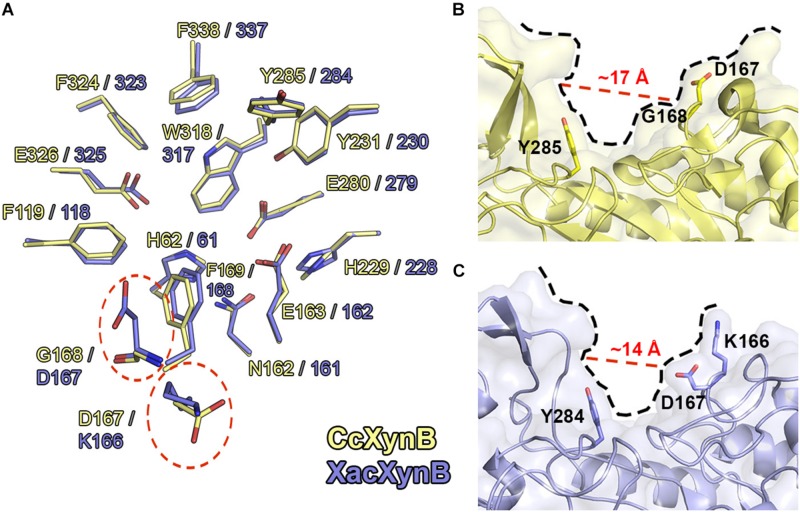
Structural comparison between CcXynB and XacXynB active sites. **(A)** Residues of the catalytic interface of XacXynB and CcXynB are superposed and shown in blue and yellow, respectively. The divergent residues from the +1 subsite are highlighted by dashed red lines. **(B)** View of the CcXynB active site, highlighting the residues from the +1 subsite (Tyr285, Gly168, and Asp167). For comparison purposes, the distance between the conserved Tyr285 and Gly168 is represented. **(C)** View of the XacXynB active site, highlighting residues from the +1 subsite (Tyr284, Asp167, and Lys166).

XacXynB showed highest structural similarity with CcXynB among the structurally characterized GH39 members (RMSD = 0.73 Å for all Cα atoms). These enzymes strictly conserve the −1 subsite and several other key residues for substrate binding including His61, Phe118, Asn161, Glu162, Phe168, His228, Tyr230, Glu279, Tyr284, Trp317, Phe323, Glu325, and Phe337 (Figure 5A). The exceptions are the residues Lys166 and Asp167, which corresponds to the positive-subsite region (Figure 5A). These are preceding the aromatic residue Phe168 directly involved in the recognition of −1 xyloside (Figure 5A). The active-site topology is clearly modified by these two residues substitutions compared to CcXynB (Figures 5B,C), suggesting that they might play a role in enzyme activity.

Kinetic characterization showed that XacXynB has a fivefold smaller K_M_ (∼1.93 mM) than CcXynB (∼9.3 mM; Corrêa et al., 2012), indicating very distinct substrate affinity among bacterial β-xylosidases from this family. As the only differences observed in the active site of these enzymes are the substitutions K166D and D167G, it would indicate a role of these residues in substrate affinity. Interestingly, the *T. saccharolyticum* GH39 β-xylosidase, which also displays a low K_M_ (Vocadlo et al., 2002), conserves a lysine (Lys164) and an acidic residue (Glu165) at the corresponding positions of Lys166 and Asp167, supporting their involvement in substrate binding.

Thus, to experimentally evaluate the role of these residues in substrate affinity, XacXynB Lys166 and Asp167 were mutated to the corresponding residues in CcXynB, an aspartate and a glycine, respectively. These mutations would mimic the GH39 β-xylosidase with lower substrate affinity. The pH and temperature dependence for catalytic activity including the k_cat_ remained similar to the wild-type enzyme (Table 2 and Supplementary Figure 3). However, both mutants presented higher K_M_ values, a twofold increase for the mutant K166D and a threefold increase for the mutant D167G (Table 2).

**TABLE 2:** Kinetic parameters of the wild-type XacXynB and the mutants K166D and D167G on 4-nitrophenyl β-D-xylopyranoside.

	**K_M_ (mM)**	**k_cat_ (min^–1^)**	**k_cat_/K_M_ (min^–1^ mM^–1^)**
WT	1.93 ± 0.25	266.0 ± 8.5	137.8
K166D	4.06 ± 0.39	219.2 ± 6.8	54.0
D167G	5.79 ± 0.35	281.0 ± 6.2	48.5

To assess the XacXynB behavior in solution, molecular dynamics simulations were performed with the WT enzyme and with the mutants (K166D and D167G). Global parameters, such as the RMSD, solvent accessible surface area (SASA) and R_g_ showed no significant difference between the WT and mutants (Supplementary Table 1 and Supplementary Figure 4), indicating that local changes are responsible for modifying the substrate affinity. SASA and R_g_ values were close to the calculated from the crystal structure and obtained experimentally by SAXS, respectively (Supplementary Table 1). Root-mean-square fluctuations (RMSF) revealed that the β-hairpin region (residues 231–249) is the most flexible region for both WT and mutants (Supplementary Figure 4).

Therefore, these substitutions at the active site have a local effect, possibly affecting the charge distribution and the positive subsite topology, favoring interactions and consequently substrate affinity. Moreover, these residues are preceding Phe168, a critical residue for the binding of the −1 xyloside and a substitution nearby could affect negatively the capacity of the enzyme to interact with the −1 xylosyl moiety.

## Discussion

The GH39 family comprises mostly β-xylosidases and α-iduronidases, with 21 characterized members and only 8 structures (CAZy, Lombard et al., 2014). Recent discoveries of polyspecific GH39 members indicate a functional and structural diversity in this family. Moreover, these enzymes are found in microorganisms living in various ecological niches including extremophilic environments, raising the interest to understand the molecular adaptations of these enzymes to be active at such harsh conditions. Previous studies suggested an important feature of this family, the C-terminal elongation in extremophilic organisms. Thus, more members of this poorly explored family need to be enzymatically and structurally characterized to validate their functions and oligomerization pattern.

In this work, a new GH39 enzyme was investigated, contributing to elucidate the structural basis for substrate affinity and to expand the concept of oligomerization as a conserved molecular strategy to gain of stability in bacterial GH39 β-xylosidases.

XacXynB is a mesophilic β-xylosidase that, like CcXynB, features a shortened C-terminus that directly implicates in the oligomerization of these proteins. Both behave as monomers in solution, whereas GH39 members from extremophilic organisms, TsXynB and GsXynB, contain the C-terminal region extended and form tetramers in solution (Yang et al., 2004; Czjzek et al., 2005). The C-terminal extension is involved in the largest contact interface that leads to oligomerization being essential for this process. Correlation between these features indicates the oligomerization as a conserved strategy in the GH39 family to enhance structural stability. By phylogenetic analysis, we verified that only GH39 from extremophilic organisms contain the C-terminal extension. Curiously, two enzymes from thermophilic species (*Caldicellulosiruptor* sp.) do not have an extended C-terminus. However, biophysical data showed that GH39 CoXylA (from *Caldicellulosiruptor owensensis*) forms dimers in solution (Mi et al., 2014), yet supporting oligomerization as an adaptive strategy for extreme environments. It is likely that in the genus *Caldicellulosiruptor* other molecular mechanisms were evolved for oligomerization and gain of stability; however, the lack of high-resolution structural data does not currently allow us to precisely predict how it occurs.

The use of oligomerization to adapt to extreme environments or to change enzyme activity is not a distinguishing characteristic of GH39 family and other CAZy families also make use of this strategy such as GH1 (Zanphorlin et al., 2016), GH5 (Lafond et al., 2016), GH10 (Santos et al., 2014), and GH32 (Sainz-Polo et al., 2013).

The unexpected occupation of the catalytic interface of XacXynB by a peptide segment from the N-terminal His-tag revealed conserved interactions with sugars moieties based on structural superpositions with other GH39 β-xylosidases in complex with xylose and analogs. According to these analyses, XacXynB active site might be able to accommodate substrates with distinct degrees of polymerization as previously observed in a GH39 member with enlarged active-site pocket (Czjzek et al., 2005). Using the capillary electrophoresis we verified the xylose release from different XOS from X2 to X6 demonstrating the capacity of XacXynB to cleave such substrates. It represents an interesting feature for industrial applications involving lignocellulose saccharification, since XOS larger than X2 that are released during hemicellulose cleavage, might competitively inhibit cellulases (Qing et al., 2010).

In addition, structural comparative analysis with CcXynB allowed the identification of non-conserved residues at the positive-subsite region that could be relevant for catalytic activity of GH39 β-xylosidases. By site-directed mutagenesis and molecular dynamics simulatioms, we revealed that the residues Lys166 and Asp167 in XacXynB can promote a gain in the substrate affinity without interfering in other catalytic properties or structural elements. These residues locally change the charge and topology of the active-site pocket, possibly increasing the interactions at the positive-subsite region with the substrate and stabilize the aromatic residue Phe168 involved in the interaction with −1 xyloside.

Together, our results demonstrate the importance of oligomerization in bacterial GH39 β-xylosidases adaptation to extreme environments and how the positive-subsite region might be redesigned to modulate substrate affinity, furthering in the mechanistic understanding of this CAZy family.

## Accession Numbers

Atomic coordinates and structure factors have been deposited in the Protein Data Bank under accession code 6UQJ.

## Data Availability Statement

The datasets generated for this study are available on request to the corresponding author.

## Author Contributions

MAM, CP, and MM designed the study, performed simulations, structural analysis, and wrote the manuscript. MAM, CP, MD, RP, FS, and JC performed the cloning, protein expression and purification, and, biochemical and biophysical experiments. GP made phylogenetic analysis and figures. MAM, CP, and CS crystallized the protein and solved the crystal and SAXS structures. All authors analyzed the results and, read and approved the final manuscript.

## Conflict of Interest

The authors declare that the research was conducted in the absence of any commercial or financial relationships that could be construed as a potential conflict of interest.
