# Supplementary material for: Exploring the Molecular Basis for Substrate Affinity and Structural Stability in Bacterial GH39 β-Xylosidases
Source: Front Bioeng Biotechnol. 2020 May 15;8:419. doi: 10.3389/fbioe.2020.00419 (PMC7242879; doi:10.3389/fbioe.2020.00419)
Supplement: Supplementary file 1 [file Data_Sheet_1.pdf]

Supplementary Material

Exploring the molecular basis for substrate affinity and structural stability in bacterial GH39 β-xylosidases

Mariana Abrahão Bueno de Morais[1, #], Carla Cristina Polo[2, #], Mariane Noronha Domingues[1], Gabriela Felix Persinoti[1], Renan Augusto Siqueira Pirolla[1], Flávio Henrique Moreira de Souza[1], Jessica Batista de Lima Correa[1], Camila Ramos dos Santos[1], Mário Tyago Murakami[1,*]

[1] Brazilian Biorenewables National Laboratory, Brazilian Center for Research in Energy and Materials, Campinas, Brazil

[2] Brazilian Synchrotron Light Laboratory, Brazilian Center for Research in Energy and Materials, Campinas, Brazil

[#]Both authors have contributed equally to this work

[*]Correspondence: Mário Tyago Murakami

mario.murakami@lnbr.cnpem.br

Keywords: glycoside hydrolase family 39, structural stability, substrate specificity, X-ray crystallography, *Xanthomonas citri*.

Supplementary Table 1. Parameters calculated from Molecular Dynamics Simulations for XacXynB WT, K166D and D167G.

	WT	K166D	D167G
RMSD (Å)	1.16 ± 0.16	1.31 ± 0.23	1.13 ± 0.13
R_g (nm)	2.40 ± 0.01<br>(SAXS 2.72 ± 0.01)	2.40 ± 0.01	2.40 ± 0.01
SASA (Å^2)	21,281 ± 312<br>(crystal structure 20,573)	21,285 ± 313	21,283 ± 270

*All parameters (averages) were calculated for 100 ns of simulation.
RMSD: Root-mean-square deviation.
R_g: Radius of gyration.
SASA: Solvent Accessible Surface Area. SASA from XacXynB WT crystal structure was calculated by PDBePISA (Krissinel and Henrick, 2007).

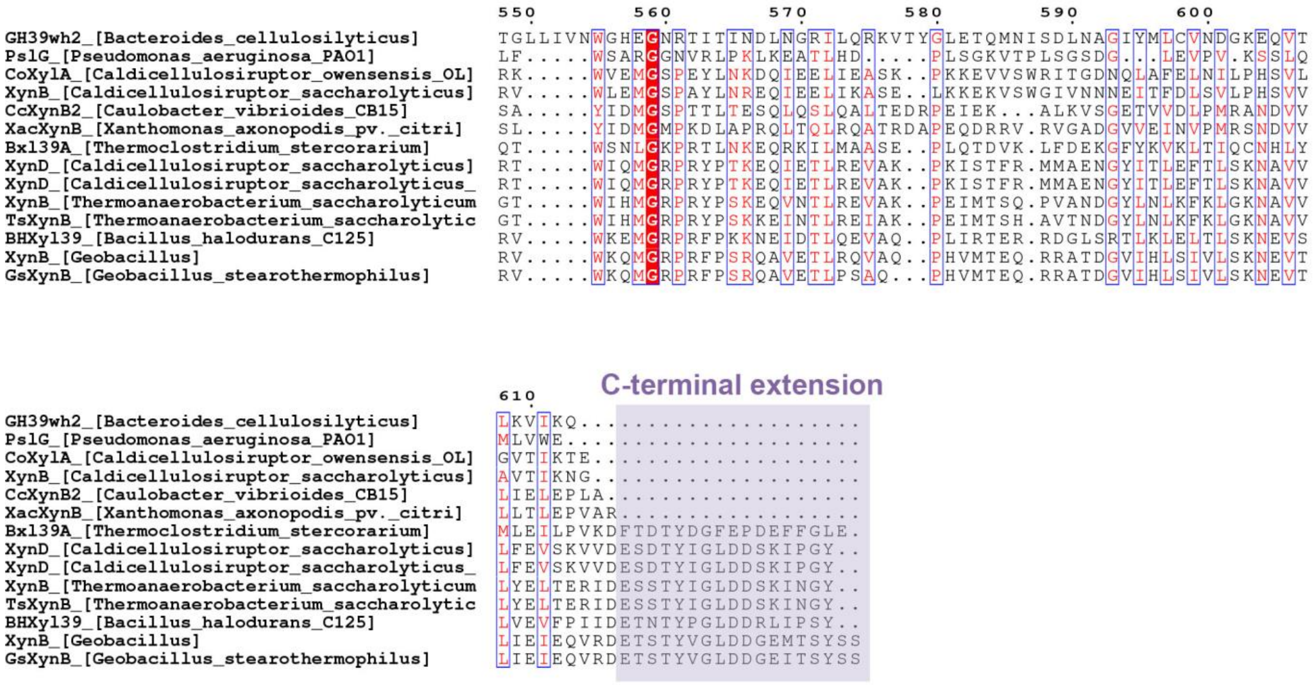

Supplementary Figure 1. Sequence alignment of XacXynB and GH39 members available in CAZy database. The C-terminal extension, present in enzymes from extremophilic organisms, is highlighted by a purple rectangle. A part of the alignment (N-terminal residues) was not represented.

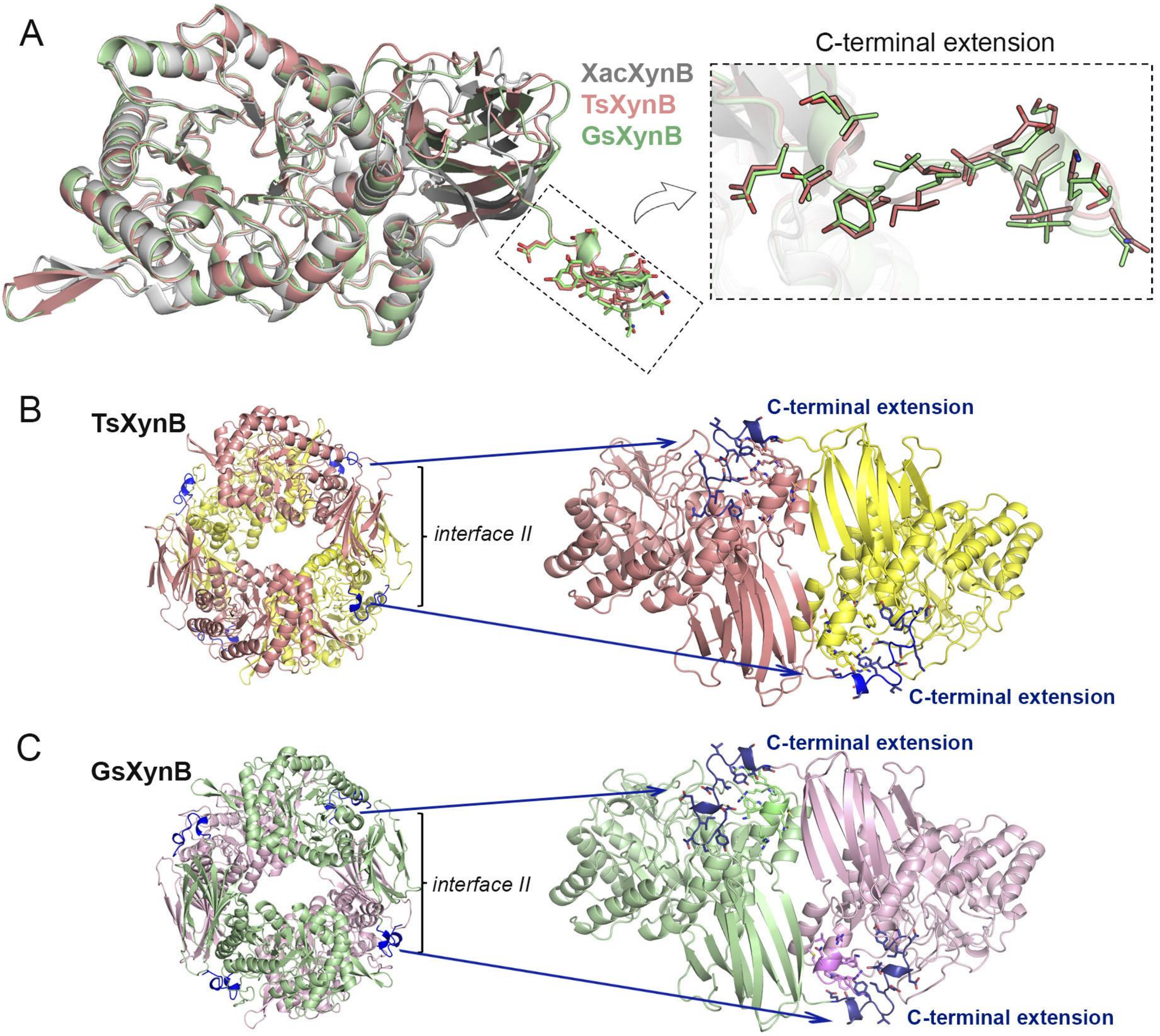

Supplementary Figure 2. Structural analysis of XacXynB and tetrameric GH39 xylosidases. (A) Structural alignment of XacXynB, TsXynB ans GsXynB, highlighting the C-terminal extension present in TsXynB and GsXynB. **(B)** Tetrameric arrangement of TsXynB **(B)** and GsXynB **(C)**, highlighting the C-terminal extensions (blue) and depicting the dimeric interfaces II.

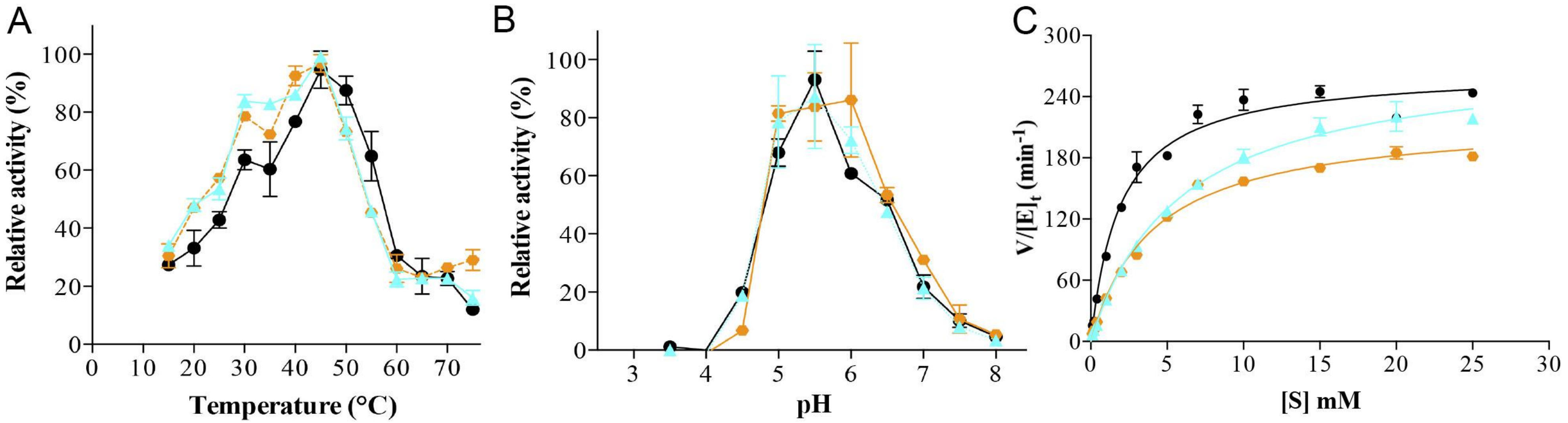

Supplementary Figure 3. Biochemical and kinetic characterization of XacXynB WT and mutants. Effects of temperature **(A)** and pH **(B)** on relative activity and kinetic curves **(C)** of XacXynB WT (black line) and mutants K166D (orange) and D167G (cyan).

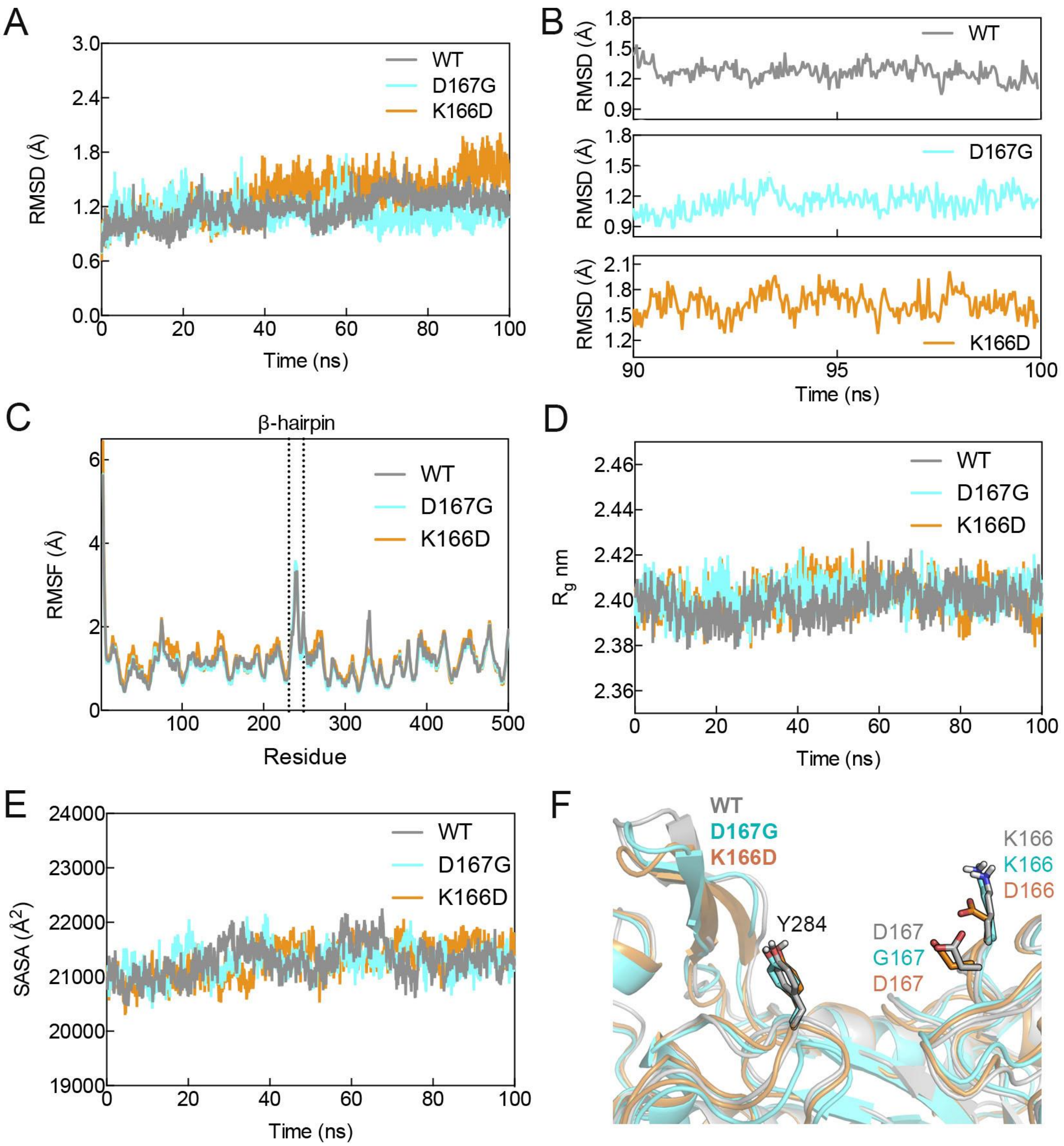

Supplementary Figure 4. Molecular Dynamics Simulations of XacXynB WT and mutants K166D and D167G. (A) Protein backbone root-mean-square deviations (RMSD) along trajectories of 100 ns. **(B)** RMSD for each system during the last 10 ns of simulation, showing that stability was reached. **(C)** Root-mean-square fluctuations (RMSF), highlighting (dashed lines) the β-hairpin region. **(D)** Radius of gyration (R_g) calculated along trajectories of 100 ns. **(E)** Solvent accessible surface area (SASA) calculated along trajectories of 100 ns. **(F)** Structures from average of representative frames taken from the trajectories of simulations with XacXynB (WT, D167G and K166D).
